# Characterization of a class II ketol-acid reductoisomerase from *Mycobacterium tuberculosis*†

**DOI:** 10.1039/d1ra08876a

**Published:** 2022-04-06

**Authors:** Ane Valera, Shan Wang, Reuben Carr, Laurent Trembleau, Hai Deng

**Affiliations:** Department of Chemistry, University of Aberdeen Aberdeen AB24 3UE Scotland UK h.deng@abdn.ac.uk; Ingenza Ltd Scotland UK

## Abstract

*Mycobacterium tuberculosis* ketol-acid reductoisomerases have been widely studied due to their metabolic importance towards development of drug-resistant bacteria treatment. We here report the biochemical characterization of a new KARI (MtKARI-II) from a *Mycobacterium tuberculosis* variant with a similar kinetic profile to class I KARIs. Phylogenetic analysis suggested that MtKARI-II is clustered into a class II KARI superfamily.

## Abstract 2

Biochemical characterization of an unusual class II KARI (MtKARI-II) from a *Mycobacterium tuberculosis* variant.

## Graphical abstract

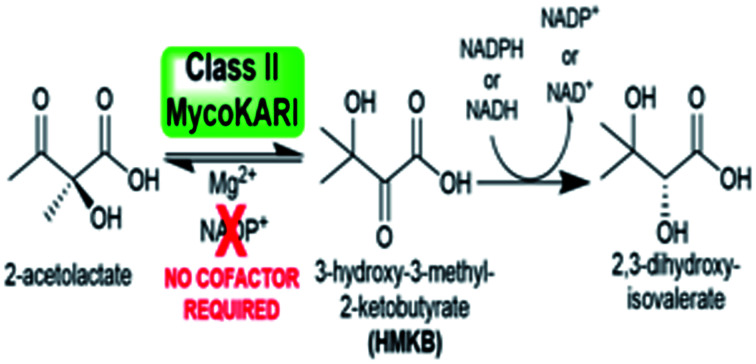

## Introduction

Tuberculosis (TB) is one of the top 10 causes of death and the leading cause from a single infectious agent. In 2019, an estimated 10 million people, including 1.2 million children, fell ill with TB worldwide.^1,2^ TB is caused by the Gram-positive pathogen, *Mycobacterium tuberculosis* (Mt) that most often affects the lungs.^3^ Currently, two frontline antibiotics, isoniazid, and rifampicin, have been used for the treatment of TB for many decades now.^4^ However, the emergence of multidrug-resistant or extensively drug-resistant Mt variants are of great concern and have further complicated treatment protocols, resulting in significantly higher mortality rates.^5^ There is a critical need to discover new, safe and cost-effective treatments for TB.

**Fig. 1:**
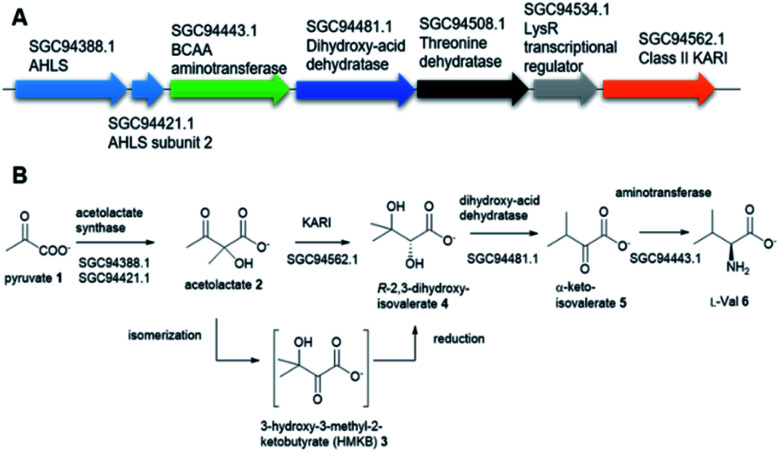
(A) The putative biosynthetic gene cluster of l-Val from a variant of *Mycobacterium tuberculosis*, encoding a putative class II KARI enzyme. (B) The proposed model of the l-Val biosynthesis starting from pyruvate 1.

The biosynthetic pathway for the branched-chain amino acids (BCAAs), such as valine, leucine and isoleucine, is present in plants, fungi and bacteria.^6^ Animals, including humans, do not have this pathway and rely on obtaining BCAAs from their diet.^1^ In this respect, the BCAA pathway makes it an attractive target for antimicrobial lead compound discovery.^7^ A typical pathway of BCAAs, such as valine, starts with an acetohydroxyacid synthase (AHAS) which acts on two molecules of pyruvate 1*via* a decarboxylation and subsequent condensation to generate acetolactate 2 (Fig. 1A).^8^ The second enzyme, ketoacid reductoisomerase (KARI) in the pathway, specifically catalyses 2*via* an unusual two-component biotransformation, a methyl migration of 2 to generate the intermediate 3-hydroxy-3-methyl-2-ketobutyrate (HMKB) 3, followed by its reduction to yield *R*-2,3-dihydroxyisovalerate 4 (Fig. 1B).^9^ Dehydration of 4 catalysed by dihydroxyacid dehydratase gives the corresponding α-ketoacid 5, the last intermediate of the pathway. Finally, the BCAA aminotransferase converts 5 into l-valine 6 (Fig. 1A).^10^

Interestingly, the BCAA pathway is crucial for the growth and survival of Mt in culture.^1^ As a result, there has been considerable interest in developing inhibitors of the enzymes involved in the BCAA pathways for anti-TB lead compound discovery. For instance, sulfometuron methyl, a commercial AHAS inhibiting herbicide has anti-TB activity in cell-based assays against the H37Rv Mt variant.^11^ KARI has also attracted considerable attention as an anti-TB drug target.^12^ KARIs are bifunctional enzymes that require Mg^2+^ as cofactor in their first half isomerization reaction and NADH or NADPH as reducing agents in the second half reduction reaction.^13*a*^ Previous studies demonstrated that HMKB 3 is the transit intermediate catalysed by the KARI enzyme (EcoKARI) from *E. coli* (Fig. 1B). It has been shown that the isomerization half of EcoKARI favours the formation of 2 over 3 in a considerable margin, suggesting that a separate isomerase would form too little of the reductase substrate to constitute an efficient system. Combining the two reactions at the single active site overcomes this difficulty, indicating that substrate isomerization and reduction are coordinated processes that are conceptually inseparable. As such 3 is regarded as a transit intermediate with little or no biological significance.^13*b*^

There are two types of KARIs. The difference between class I and class II KARIs relies on their length and on the organisms in which this enzyme is expressed.^14^ Class I KARIs present a shorter aminoacidic sequence and are commonly found in microorganisms in comparison to class II KARIs mostly present in plants which are ∼140 AA residues longer than the class I counterparts.^6^ Due to its metabolic importance, KARI from Mt has extensively been studied and its structure has been solved.^14^ MtKARI is a typical class I KARI with a sequence length of 337 amino acid residues which only utilize NADPH as the reducing agent.^1^

Recent advances of next-generation genome sequencing technology have witnessed an improved understanding of the genetic diversity of Mt and Mt variants which translates into significant differences in immunogenicity and virulence.^15^ Such genetic diversity among Mt strains is thought to be partly because of horizontal gene transfer (HGT) from unrelated bacterial species.^16^ Indeed, a recent phylogenetic analysis^17^ strongly suggested that the gene encoded for polyadenylate polymerase in the Mt variant 2926STDY5723586, an enzyme catalysing polyadenylation of RNA 3′-ends for the bacterial RNA degradation pathway, is likely to be originated from the potential donor strain, *Morganella morganii*, a γ-proteobacterial opportunistic pathogen found in intestinal tracts of humans, mammals, and reptiles as normal flora.

This is also the case for the gene encoding for KARI. While the majority of Mt and Mt variants contain class I KARIs, the Mt strain 2926STDY5723586 possesses an unusual putative KARI (MtKARI-II, access no SGC94562.1) with an annotated sequence length of 491 amino acid residues. Examination of the corresponding genetic surrounding allowed the identification of three genes encoding putative AHAS (SGC94388.1), BCAA aminotransferase (SGC94443.1), and dihydroxyacid dehydratase (SGC94481.1), respectively (Fig. 1A). Interestingly, MtKARI-II contains identical sequences at DNA level (100% DNA sequence identity) and amino acidic level (100% AA identity) with the one from the variant of *Morganella morganii* N18-00103, suggesting a possible HGT between two species.^16^

Here, we report the characterization of MtKARI-II from the Mt variant strain 2926STDY5723586. Our kinetic analysis demonstrates that MtKARI-II displays a similar kinetic profile in comparison to the typical class II KARI (EcoKARI) from *E. coli*. We also demonstrate that MtKARI-II efficiently converts the synthetic HMKB 3 into either acetolactate 2*via* isomerization in the absence of NADPH or *R*-2,3-dihydroxyisovalerate 4*via* reduction by NADPH, demonstrating a two-step enzymatic process. Interestingly, MtKARI-II can utilise both NADPH and NADH as the reducing agents, a clear difference to the MtKARI previously studied. Finally, our phylogenetic analysis suggests that MtKARI-II diverges from the class I KARIs, including MtKARI and is clustered into the class II KARIs.

## Results and discussion

To validate the function of the putative gene product (SGC94443.1) in the Mt variant strain 2926STDY5723586, we then set out to overexpress the gene in *E. coli* BL-21 (DE3). The recombinant MtKARI-II was expressed as an N-terminal His_6_-protein and the soluble protein was purified to near homogeneity with the expected molecular weight (54 kDa) as indicated in the SDS page analysis (Fig. S1†). Incubation at 37 °C of MtKARI-II (65 μM) with MgCl_2_ (10 mM), NADPH (0.22 mM) in 0.1 M Tris/HCl buffer (pH = 8.0) and various concentrations of 2 provided compound 4, the molecular identity of which was confirmed by UHPLC-high resolution-electrospray ionisation-mass spectrometry (MS) (UHPLC-HR-ESI-MS), indicating that the putative MtKARI-II is indeed a KARI enzyme (Fig. S2†).

The activity of MtKARI-II was estimated from NADPH oxidation by 2 by measuring the absorption at 340 nm using an extinction coefficient of 6220 M^−1^ cm^−1^. By this assay, MtKARI-II was found to have catalytic parameters that are in good agreement with those reported for EcoKARI with *k*_cat_ = 2.23 ± 0.1 s^−1^, *K*_M_ = 250 ± 30 μM,^18^ MtKARI with *k*_cat_ = 1.4 ± 0.02 s^−1^, *K*_M_ = 110 ± 4 μM (ref. 1) (Table S1†) and MtH37Rv (Mtb-Rv) and Mt H37Ra (Mtb-Ra) strains with a *K*_M_ = 110 ± 4 μM.^18^ MtKARI-II also displayed similar catalytic parameters to that found for KARIs from other microorganisms, suggesting a conserved reaction mechanism (Table S1†). Interestingly, MtKARI-II could use NADH as the reducing agent, albeit less efficiently. The catalytic parameters for 2 are *k*_cat_ = 71.5 ± 4.77 s^−1^, *K*_M_ = 488.76 ± 57.04 μM, suggesting that MtKARI-II has a cofactor preference for NADPH rather than NADH. While most KARIs prefer NADPH as a cofactor, a few bacterial KARIs were also reported to utilize both NADPH and NADH as reducing agents. There has been considerable interest in exploring the biocatalytic potential of NADH-dependent KARIs as they are desirable in industrial applications including anaerobic fermentation to produce branched-chain amino acid or biofuels.^19^ The fact that MtKARI-II is a new NADH/NADPH bi-cofactor-dependent KARI suggested that this Mt variant may possess a considerable degree of evolutionary advantages because living cells usually contain higher levels of NADH.

KARIs have been proposed to be bifunctional enzymes with two catalytic sites, one for the isomerization reaction and the second for the reduction step. To confirm whether MtKARI-II follows a similar process, we set out to synthesize HMKB 3. Various synthetic approaches have been reported in the literature. One attempt involves the bromination of commercially available ethyl 3-methyl-2-oxobutyrate 7 with Br_2_, followed by hydrolysis providing 3 (Scheme S1†).^20^ The second method starts with the esterification of the commercially available dichloroacetyl chloride 8, followed by a Darzens condensation with acetone to form the oxirane ester intermediate 10, which is hydrolysed to lead to the desired monosodium salt 3 in a straightforward manner.^21^

We synthesized HMKB using the second approach above following a protocol described in a patent as the synthetic steps appeared straightforward with an excellent reported yield. After the optimization of the protocols, we were able to produce compound 9 in good yield and purity (Fig. S3–S8†). Unstable epoxide intermediate 10 was produced at low temperatures and directly submitted to hydrolysis in a one-pot reaction to provide a solution of desired compound 3 (Fig. S7 and S8†). Analysis of the solution by UHPLC-HR-ESI-MS analysis indicated the presence of an ion corresponding to 3 (Fig. S9†).

**Fig. 2:**
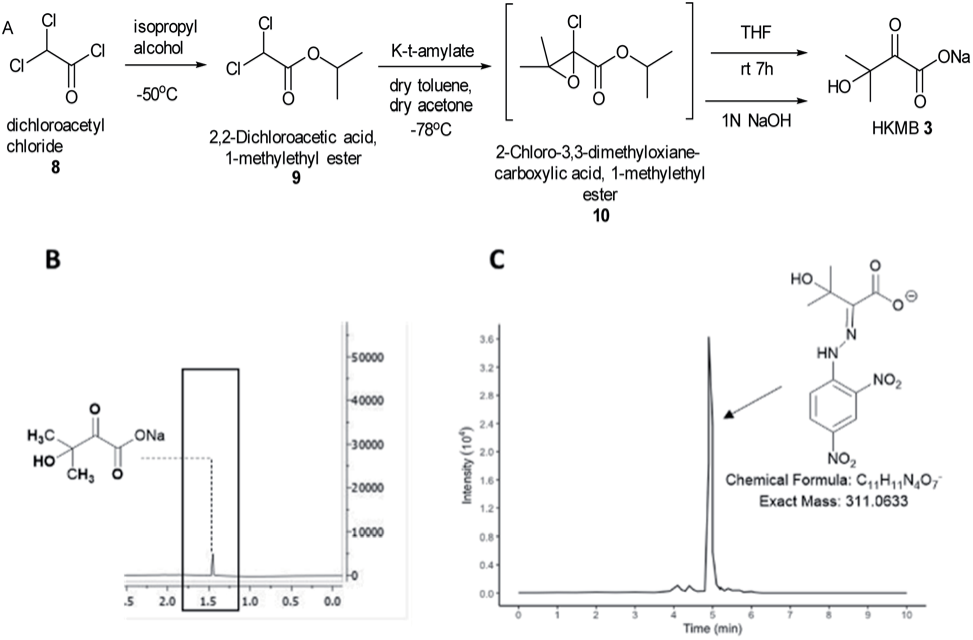
(A) An optimised synthesis scheme towards HMKB 3 starting from dichloroacetyl chloride 8, *via* isopropyl 2-chloro-3,3-dimethyloxianoate 10 as a transit intermediate. (B) ^1^H-NMR of 3 confirming the presence of the compound. (C) Extracted ion chromatogram (EICs) from LC-MS analysis of 3-DNPH.

To further confirm its identity, the synthetic product was then subjected to the derivatization with 2,4-dinitrophenylhydrazine (DNPH) (Fig. S10†). The ion corresponding to the 3-DNPH adduct was observed in the UHPLC-HR-ESI-MS spectrum (Fig. 2).

Interpretation of ^1^H and ^13^C NMR spectra demonstrated the presence of 3 (sodium salt) with a minute amount of impurity (Fig. 2). Attempts to isolate the corresponding carboxylic acid product proved difficult but the salt form of 3 was adequate for the study of MtKARI-II. To this end, we determined the concentration of salt 3 in solution by ^1^H-NMR using glycerol as internal standard. By integration of the relevant NMR signals, we estimated a concentration of 1.4 × 10^−4^ M.

**Fig. 3:**
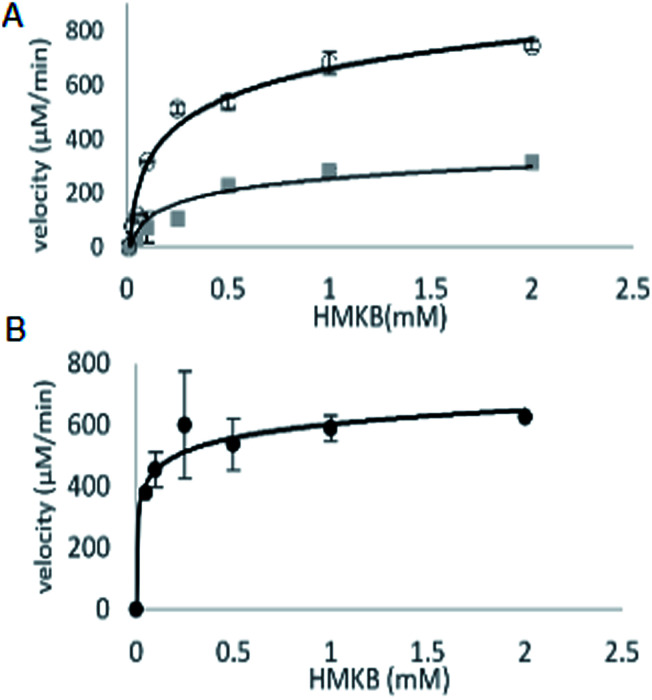
(A) The kinetic curves of HMKB for reductase cycle using NADPH (circle dots) and NADH (square dots). (B) Kinetic curve for reverse isomerase cycle of MtKARI-II with HMKB as substrate.

With synthetic 3 available, the kinetic study of MtKARI-II enzyme was performed. We first measured the reductive cycle by monitoring NADPH consumption. Incubation of MtKARI-II (65 μM) with NADPH (0.22 mM) in 0.1 M Tris/HCl buffer (pH = 8.0) and various concentrations of 3 provide the production of 4. The kinetic constants involving the measurement of NADPH oxidation by 3 were the values of *k*_cat_ = 201.17 ± 61.39 s^−1^ and *K*_M_ = 301.30 ± 7.7 μM (Fig. 3). A similar *K*_M_ for HMKB was also found in the KARI from *E. coli* K12 (*K*_M_ = 0.27 mM), suggesting that, apart from showing structural homology, MtKARI-II and *E. coli* K12 KARI presents similar affinity for 3. MtKARI-II was also incubated with NADH and various concentrations of 3 for *R*-2,3-hydroxyisovalerate formation with the calculated kinetic values of *k*_cat_ = 79.25 ± 32.88 s^−1^ and a *K*_M_ = 556.15 ± 137.8 μM, indicating that, even if MtKARI-II can accommodate NADH, NADPH is preferred for the reductive cycle. Considerable efforts have been made to engineer and switch NADPH-dependent KARI to be switched into NADH-utilizing enzymes.^22^ Our biochemical analysis demonstrated that MtKARI-II is a new NADPH/NADH bi-cofactor-utilizing KARI from the Mt variant 2926STDY5723586.

To investigate the isomerization cycle on HMKB 3, we developed a discontinuous colorimetric assay of the production of 2. 2 is known to be decarboxylated to acetoin under acidic conditions, later formed acetoin will be reacted to diacetyl under alkali conditions. Diacetyl can be coupled with creatine and α-naphthol displaying a bright red colour at 540 nm due to the reaction between the diacetyl and the guanidino group from the creatine^23^ forming a heterocycle product (Fig. S11†). We first established a standard curve of the commercially available acetolactate-derived acetoin adduct (Fig. S12†). After acid treatment, the enzymatic mixture containing acetoin and diacetyl was derivatized with DNPH. LC-MS analysis demonstrates the presence of two ions with *m*/*z* values of 266.0657 and 267.0735 in the negative mode, corresponding to diacetyl-DNPH and acetoin-DNPH, respectively. We then derivatise the enzyme reaction mixture with creatine and α-naphthol. The identity of diacetyl-creatine heterocycle was confirmed by UPLC-ESI-MS (Fig. S13†).

The steady-state parameters of the isomerization cycle by MtKARI-II on 3 were determined by the nonlinear fitting of Michaelis–Menten equation. The assays were conducted using a range of 3 (2–0.05 mM). The apparent *K*_M_ value for 3 was calculated to be 55.54 ± 12.50 μM (Fig. 3).

**Fig. 4:**
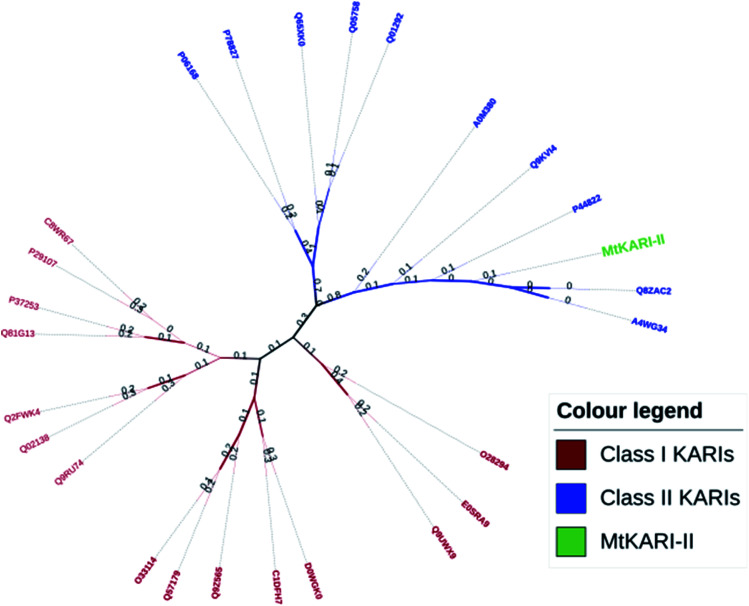
Phylogenetic tree analysis. Maximum likelihood method and JTT matrix-based models were applied for the analysis.

Unlike other KARIs from Mt strains, this MtKARI-II contains 491 amino acid residues in its sequence, making it an interesting issue to investigate the phylogenetic relationship of MtKARI-II with other KARI enzymes. Our phylogenetic analysis of MtKARI-II and its analogues suggested that MtKARI-II belongs to the cluster of class II KARIs among other characterized KARI enzymes (Fig. 4). Protein modelling in Phyre server^24^ suggested that this putative MtKARI-II has high structural homologue (100% confidence and 86% i.d.) (Fig. S8†) to the typical class II KARI from *E. coli* (PDB no. 1YRL) with the conserved amino acid residues.

## Conclusions

We have biochemically characterized a new class II KARI from a variant of Mt. Biochemical analysis indicated that this enzyme catalyses its native substrate acetolactate towards *R*-2,3-dihydroxyisovalerate. We also developed an optimised synthesis towards HMKB 3, which was shown to be the substrate for both isomerization and reduction reaction cycles of this MtKARI-II. Our data showed that MtKARI-II is an NADPH/NADH bi-cofactor utilizing enzyme, making it a potential candidate for use in metabolic engineering or industrial applications under anaerobic conditions.

## Conflicts of interest

The authors declare no conflicts of interest.

## Supplementary Material

RA-012-D1RA08876A-s001
