# Supplementary material for: Characterization of a class II ketol-acid reductoisomerase from Mycobacterium tuberculosis
Source: RSC Adv. 2022 Apr 6;12(17):10540–4. doi: 10.1039/d1ra08876a (PMC8985424; doi:10.1039/d1ra08876a)
Supplement: RA-012-D1RA08876A-s001 [file RA-012-D1RA08876A-s001.pdf]

Electronic Supplementary Material (ESI) for RSC Advances.
This journal is © The Royal Society of Chemistry 2022

Supplementary Information

Characterization of a new class II ketol-acid reductoisomerase from *Mycobacterium tuberculosis*

Ane Valera[1], Shan Wang[1], Reuben Carr[2], Laurent Trembleau[1], Hai Deng[1]

[1] Department of Chemistry, University of Aberdeen, Aberdeen, AB24 3UE, Scotland, United Kingdom

[2] Ingenza Ltd Scotland, United Kingdom

Contents

1. Materials and methods 2
1.1 General Experimental Procedures 2
1.2 Protein expression of recombinant *Mt*KARI-II 2
1.3 Purification of His_6-*Mt*KARI-II 2
1.4 Enzymatic kinetic studies 2
1.4.1 Reductosiomerase activity 2
1.4.2 Reductase activity 3
1.4.3 Reverse isomerase activity 3
1.4.4 Kinetics calculations 3
2. Synthesis of sodium 3-hydroxy-3-methyl-2-oxobutanoate (HMKB) 3 4
2.1 Literature synthesis attempts for HMKB **3** 4
2.2 Synthesis of isopropyl dichloroacetate **9** 4
2.4 Synthesis of 2-Chloro-3,3-dimethyloxianecarboxylic acid, 1-methylethyl ester **10** 4
2.5 Synthesis of sodium 3-hydroxy-3-methyl-2-oxobutanoate **3** 5
3. Supplementary Figures 6
4. Supplementary table 20
5. References 21

1. Materials and methods

1.1 General Experimental Procedures

All the chemicals and reagents employed were purchased at the highest commercial quality from Sigma Aldrich, except that (S)-α-acetolactic acid potassium salt was purchased from Toronto Research chemicals (TRC). All chemicals and reagents were used without further purification. Synthetic genes were provided by Genscript Biotech.

Proton nuclear magnetic resonance (^1H NMR) spectra and carbon nuclear magnetic resonance (^{13}C NMR) were recorded on Bruker AVANCE III HD 400 MHz (Ascend™ 9.4 Tesla) or Bruker AVANCE III HD 600 MHz (Ascend™ 14.1 Tesla) with Prodigy TCI™ cryoprobe. Chemical shifts for protons are reported in parts per million scale (δ scale) downfield from tetramethylsilane (TMS) and are referenced to residual protium in the NMR solvents, CD_3OD δ3.31 and $CDCl_3$ δ7.26 (Goss Scientific). Data are presented as follows: chemical shift, abbreviations for NMR data multiplicity are s = singlet, d = doublet, t = triplet, q = quartet, m = multiplet, br = broad, integration and coupling constant in Hz. High-Resolution Electrospray Ionisation Mass Spectrometry (HR-ESIMS) was measured by LTQ Orbitrap Thermo Scientific MS system coupled to a Thermo Instrument HPLC system (Accela PDA detector, Accela PDA autosampler and Accela Pump). The absorbance readings were measured using UV-vis spectrophotometer (Jenway 6300/05/20D).

1.2 Protein expression of recombinant *Mt*KARI-II

The synthetic pET28-mycoKARI plasmid was transformed into *E. coli* BL21 (DE3) competent cells by heat shock. Single colonies were grown overnight in Luria-Bertani (LB) broth (1% tryptone, 0.5% yeast extract, 1% NaCl) (5 mL) containing kanamycin (50 µg/mL). The overnight culture was transferred to fresh LB medium (500 mL) supplemented with kanamycin (50 µg/mL) and cultivated at 37 °C until the cell density reached an OD_{600} of 0.6. IPTG was added to a final concentration of 1 mM to induce protein expression. Cells were grown for 16 hs at 16 °C and then harvested by centrifugation at 4 °C.

1.3 Purification of His_6-*Mt*KARI-II

The cells pellets were resuspended in ice-cold lysis buffer (PBS buffer with 10 mM imidazole, pH 7.4), and further disrupted by Ultrasonic Homogenizer JY92-IIN. Then the supernatant of cell debris was loaded onto Ni-NTA-affinity column. Bound proteins were eluted with the same PBS buffer containing 200 mM imidazole. The desired elution fractions were combined and concentrated using a Centrifugal Filter Unit (Millipore). The purity and size of the proteins were confirmed by sodium dodecyl sulphate polyacrylamide gel electrophoresis (SDS-PAGE) analysis. Protein concentrations measurements were performed by the Colibri microvolume spectrometer (Titertek Berthold) using protein's molecular weight and extinction coefficient.

1.4 Enzymatic kinetic studies

1.4.1 Reductosiomerase activity

Reductoisomerase activity measurements for the enzyme were performed as described by Tyagi *et al.*[1] at room temperature in 0.1 M PBS buffer (pH 7.4) containing 0.22 mM NADPH, 10 Mm $MgCl_2$ and various concentrations of (S)-α-acetolactic acid potassium salt **2** in a total volume of 250 µl. The consumption of NADPH was measured by the change in absorbance at 340 nm monitored by a Genova (JENWAY) spectrophotometer.

1.4.2 Reductase activity

Reductase activity of *Mt*KARI-II was carried out with the same procedure as for the reductoisomerase activity [1] except chemically synthesised **3** used as the substrate. NADPH consumption was measured by the changes in absorbance at 340 nm monitored by a Genova (JENWAY) spectrophotometer.

1.4.3 Reverse isomerase activity

Reverse isomerase activity of *Mt*KARI-II was measured in a water bath at 37 ºC in 0.1 M potassium phosphate buffer (pH 8.0) containing 10 mM $MgCl_2$ and 100 μM HMKB. After 30 min, the reaction was stopped by addition of 0.5% (v ⁄ v) H_2SO_4 [1]and 2-acetolactate was estimated using creatine and a-naphthol method [2] by measuring the formed acetoin after one hour incubation at 530 nm monitored by Genova (JENWAY) spectrophotometer.

1.4.4 Kinetics calculations

Nonlinear regression of obtained absorbance data was fitted into the Michaelis–Menten equation, which allowed the calculation of the values for K_m and V_{max} values.

2. Synthesis of sodium 3-hydroxy-3-methyl-2-oxobutanoate (HMKB) 3

2.1 Literature synthesis attempts for HMKB **3**

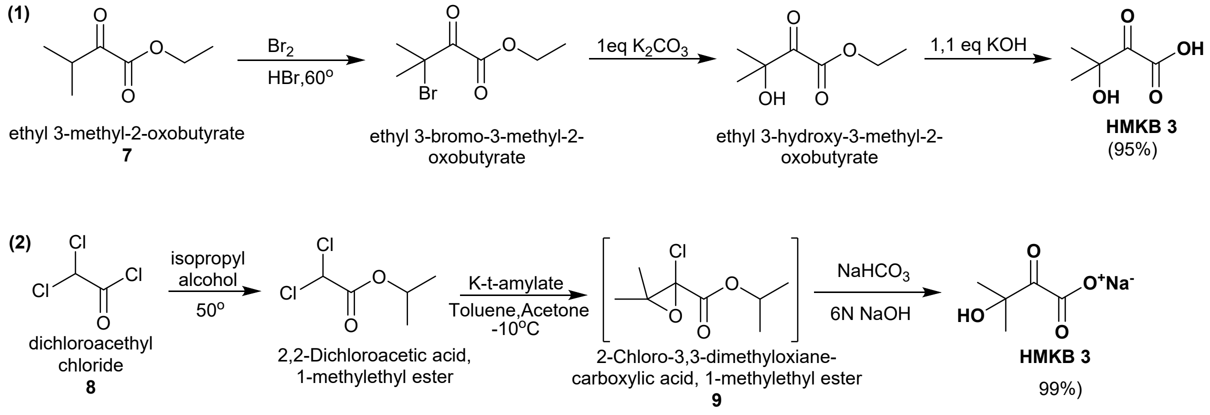

Scheme S1. Synthesis attempts for the synthesis of **3**. (1) bromination of ethyl-3-methyl-2-oxobutyrate **7**, followed by hydrolysis to yield **3**. [3] (2) estification of dichloroacetyl chloride **8**, followed by rearrangement under basic conditions to provide **3**. [4]

2.2 Synthesis of isopropyl dichloroacetate **9**

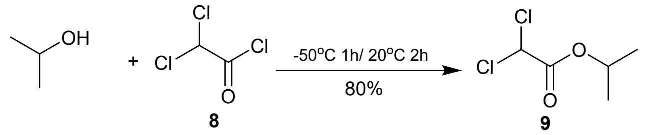

8 **9**

Isopropyl alcohol (IPA) (20g,0.27mol, 2 eq) was added to the flask (50 ml) and $CHCl_2COCl$ **8** (16.2g, 0.135mol, 1 eq) was added dropwise in an acetone/dry ice bath (-50°C). After an hour, the mixture was warmed to rt and left to stir for another two hrs. The solvent was washed with brine (25 ml) and water (25 ml). EtOAc (25 ml) were added for layer separation and compound extraction. The EtOAc layer was washed with 5% $NaHCO_3$ (25 ml), and then brine (10 ml). The obtained organic layers were dried over $MgSO_4$. The remaining EtOAc was distilled to yield the ester, **9**.

^1H NMR (400 MHz, DMSO-d_6) δ 6.79 (s, 1H), 4.03 (m, *J* = 7.1 Hz, 1H), 1.27 (d, *J* = 6.3 Hz, 7H). The other peaks are ethyl acetate solvent traces. ^{13}C NMR (400 MHz, Chloroform-*d*) δ 164.00, 71.90, 64.61, 21.32. IR: (C=O) 1758 cm^{-1}. RESIMS calculated for $C_5H_8Cl_2O_2^+$ $[M+H]^+$ 170.0170, found 170.0174. Yield **9**: 80.30% (18.63 g)

2.4 Synthesis of 2-Chloro-3,3-dimethyloxianecarboxylic acid, 1-methylethyl ester **10**

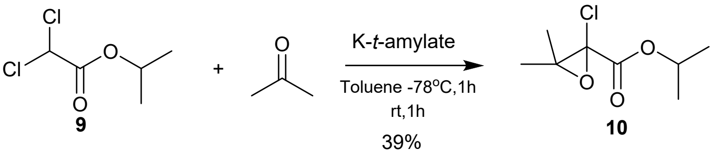

9 **10**

Compound **9** (2 g, 0.011 mol) was dissolved in a mixture of dry acetone (0.8ml, 0.01 mol) and dry toluene (0.9 ml, 0.01mol) in a three-necked flask (25 ml) under argon protection. The reaction mixture was then cooled to -78°C in the acetone/dry ice bath. Potassium K-*t*-amylate

(1.44g, 0.011mol dissolved in 2 ml dry toluene) was slowly added under stirring. After an hour, the mixture was warmed to rt and left to stir for another one hr. The mixture was quenched with brine (2.5 ml). the organic layer was washed with 50% brine (2.5 ml) and brine (2.5 ml) and dried with $MgSO_4$ before evaporation under reduced pressure.

^{1}H NMR (400 MHz, DMSO-d_6) δ 5.67 (s, 1H), 5.02 (hept, J = 6.3 Hz, 1H), 1.43 (s,6H), 1.28(d, J=6.3 Hz,3H),1.15(s,3H).^{13}C NMR (400 MHz, Chloroform-*d*) 19.99, 21.52, 64.60, 70.83, 80.03,164.14 .IR: (C=O) x2: 1718.7,1739.1 cm^{-1}, (OH) 3506.1 cm^{-1}. RESIMS calculated for $C_8H_{13}Cl_2O_2^+$ [M+H] $^+$ 193.0553, found: 193.0557. Yield **10**: 39.11% (0.88g).

2.5 Synthesis of sodium 3-hydroxy-3-methyl-2-oxobutanoate **3**

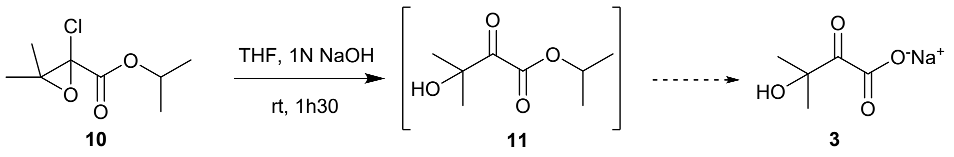

To a solution of compound **10** (0.6 g, 0.0031 mol) in tetrahydrofuran (30 ml, 0.36mol), 1 N NaOH (9 ml, 0.339 mol) was added. After 1.5-hr stirring, the reaction mixture was washed with EtOAc (10 ml) twice. The aqueous phase was collected and freeze dried.

^{1}H NMR (400 MHz, DMSO-d_6) δ 1.45 (s, 6H). ^{13}C NMR (400 MHz, DMSO-d_6) δ 209.95,177.34,25.83. HRESIMS calculated for $C_5H_8O_4^-$ [M+H]$^-$131.0350, found 131.0355. Yield **3**: 72.46% (0.5g).

3. Supplementary Figures

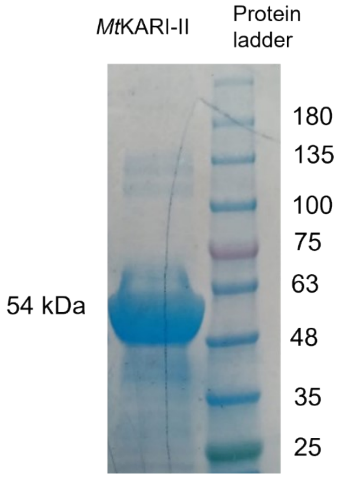

Figure S1. SDS-PAGE Analysis of recombinant N-His_6-tagged *Mt*KARI-II.

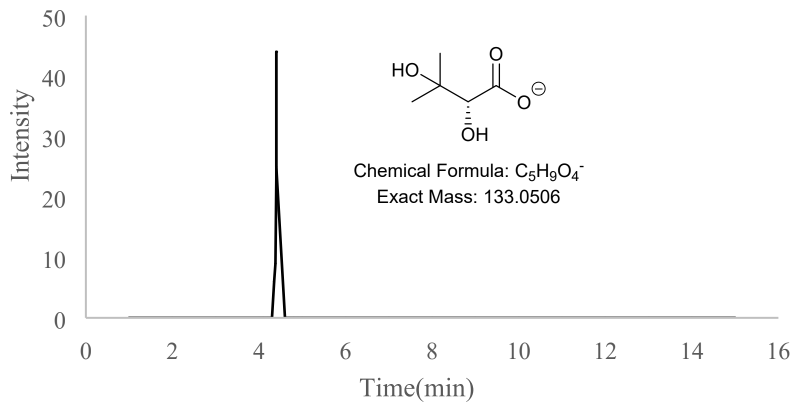

Figure S2. UHPLC-HR-ESI-MS analysis of the production of *R*-2,3-dihydroxyisovalerate (*m*/*z* 133.0516 [M-H], exact mass: 133.0505) in the assay of *Mt*KARI-II.

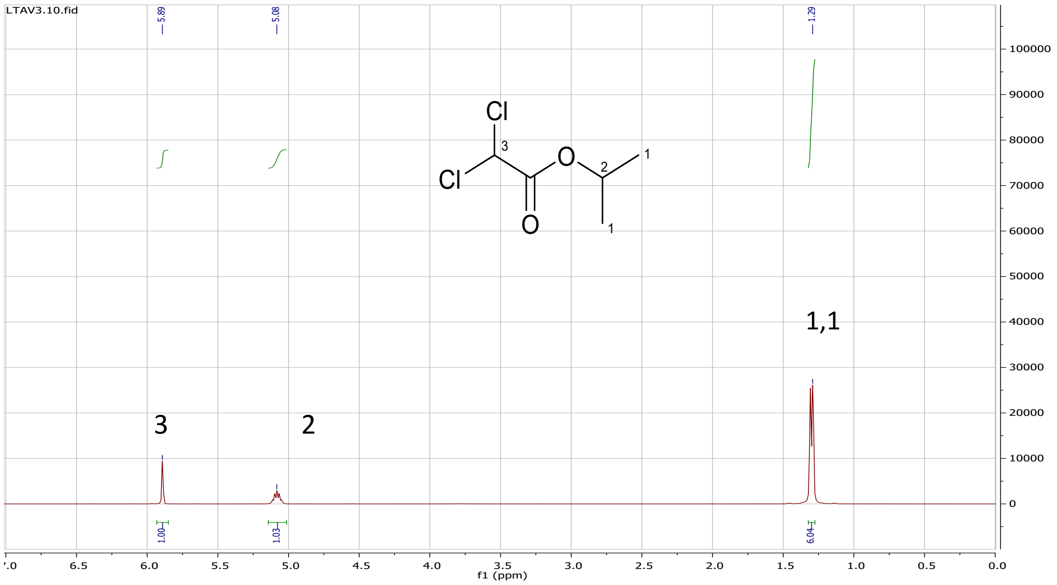

Figure S3. ^{1}H-NMR of isopropyl dichloroacetate **9** (600MHz, DMS0-d_6).

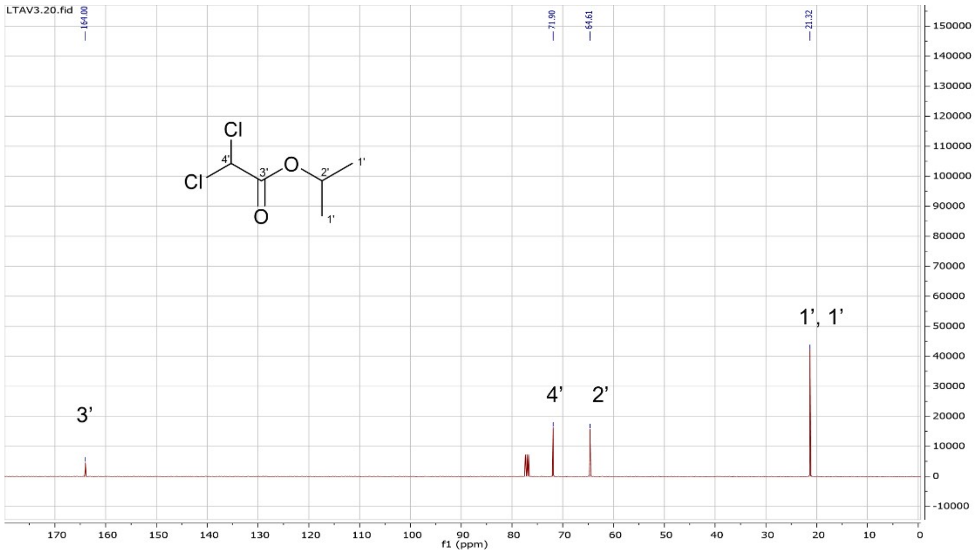

Figure S4. [13] C-NMR of isopropyl dichloroacetate **9** (400MHz, Chloroform-*d*)

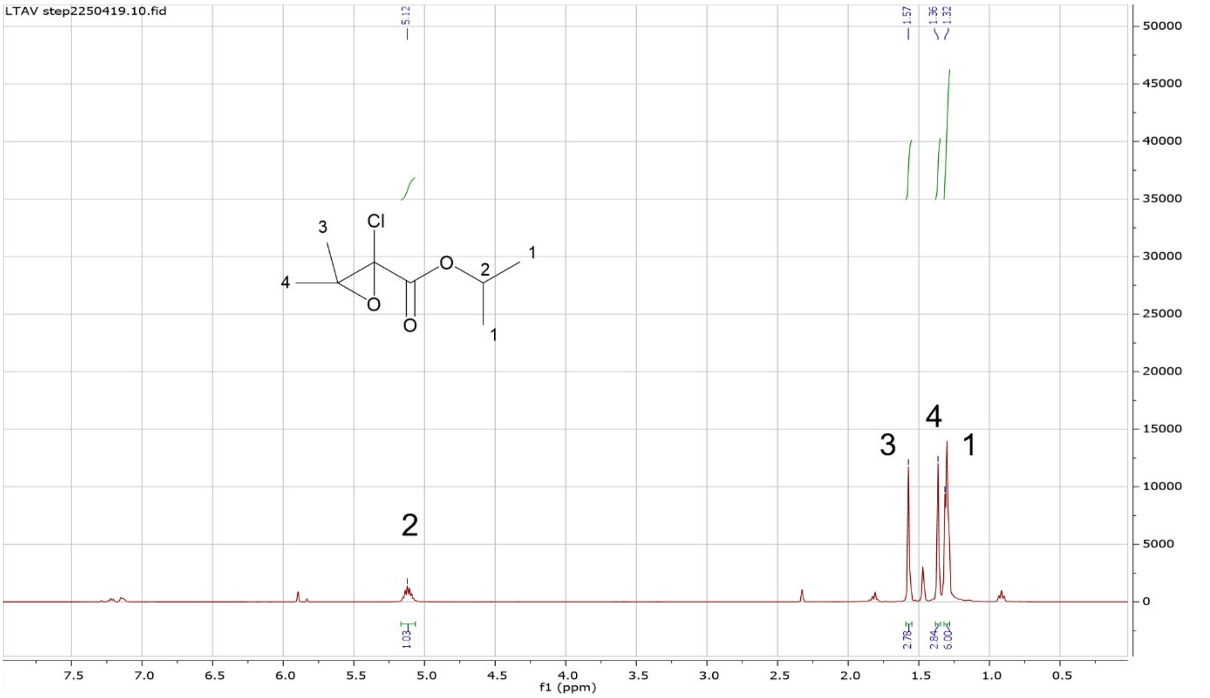

Figure S5. ^{1}H-NMR of 2-chloro 3,3 dimethyloxianecarboxylic acid, 1-methylethyl ester **10** (600MHz, DMS0-d_6) (Compound **10** was found to be sensible to water, often giving the hydrolysed intermediate **11**. The NMR spectrum of the crude compound is shown above containing some unidentified impurities).

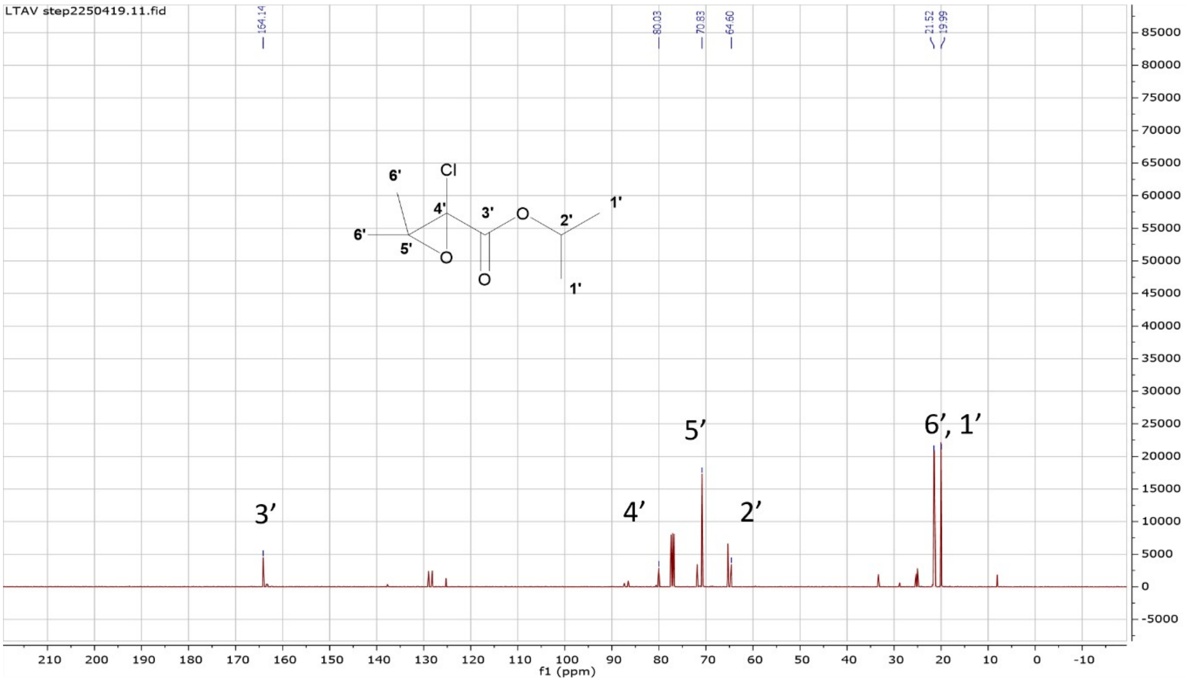

Figure S6.[13] C-NMR of 2-chloro-3,3 dimethyloxianecarboxylic acid, 1-methylethyl ester **10** (600MHz, DMSO-d_6) (Compound **10** was found to be sensible to water, often giving the hydrolysed intermediate **11**. The NMR spectrum of the crude compound is shown above containing some unidentified impurities).

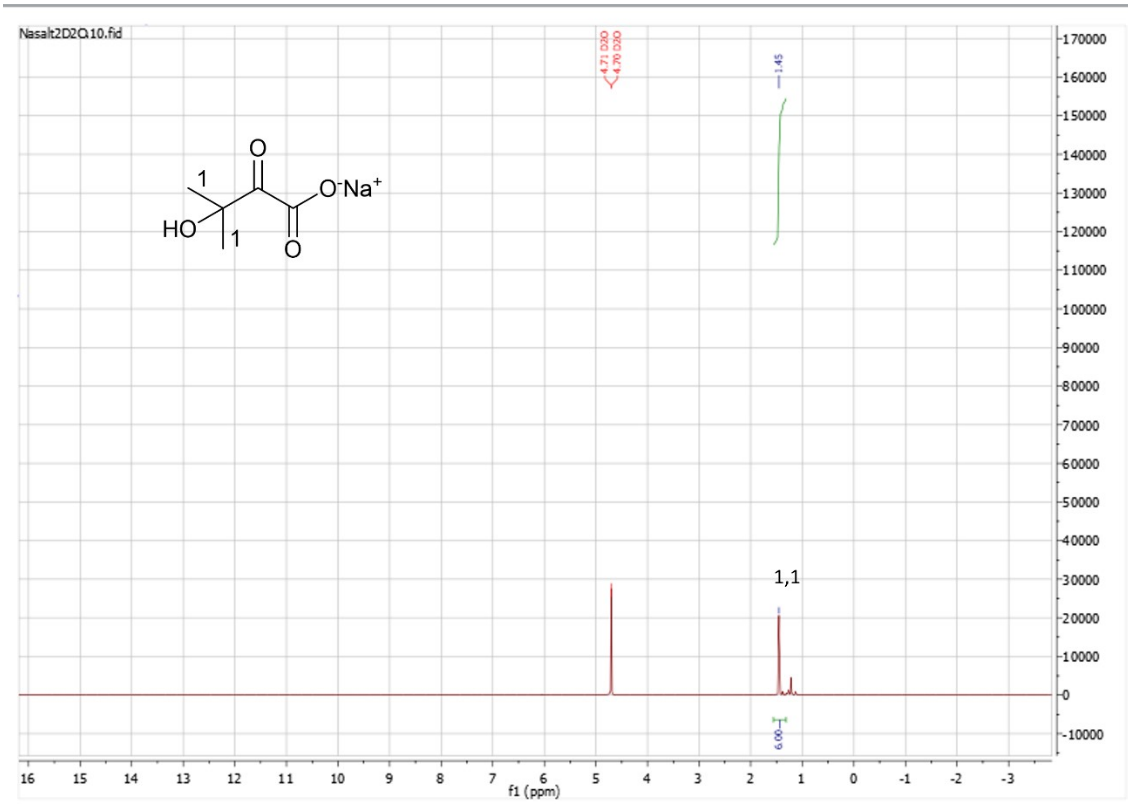

Figure S7. ^{1}H-NMR of sodium 3-hydroxy-3-methyl-2-oxobutanoate **3** (600MHz, DMSO-d_6).

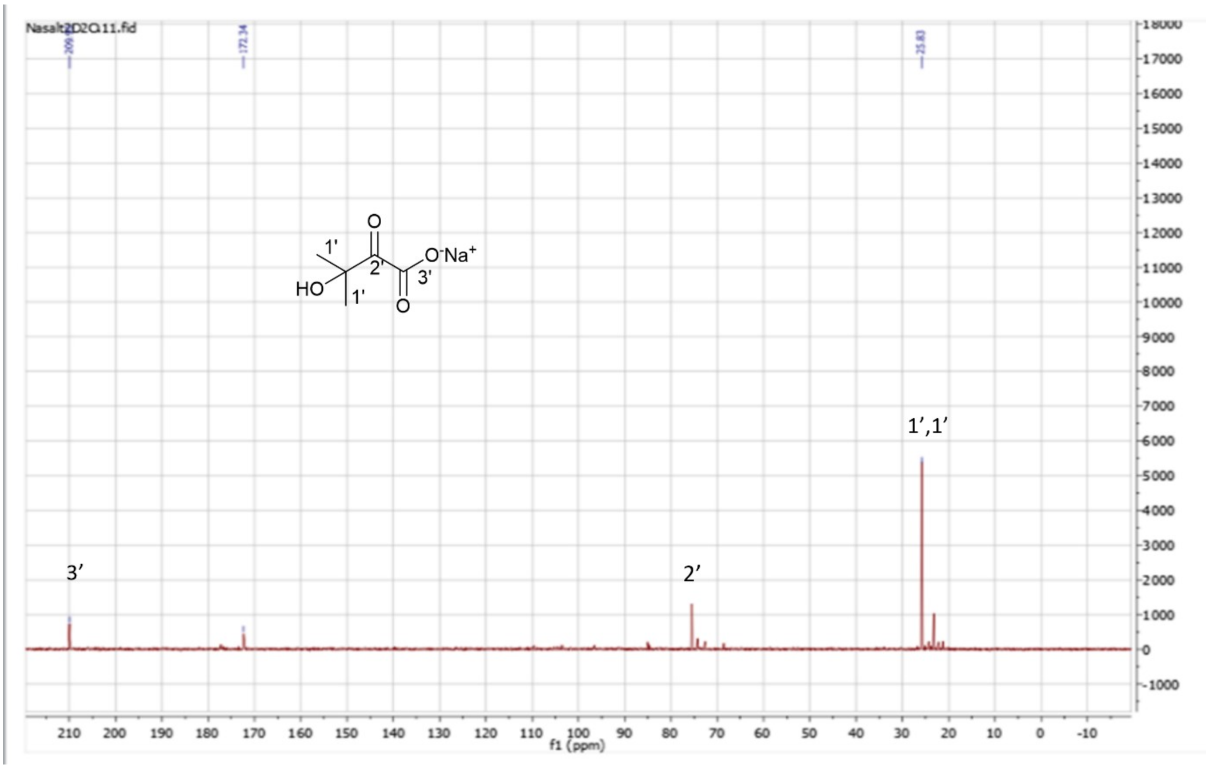

Figure S8. [13] C-NMR of sodium 3-hydroxy-3-methyl-2-oxobutanoate **3** (600MHz, DMSO-d_6).

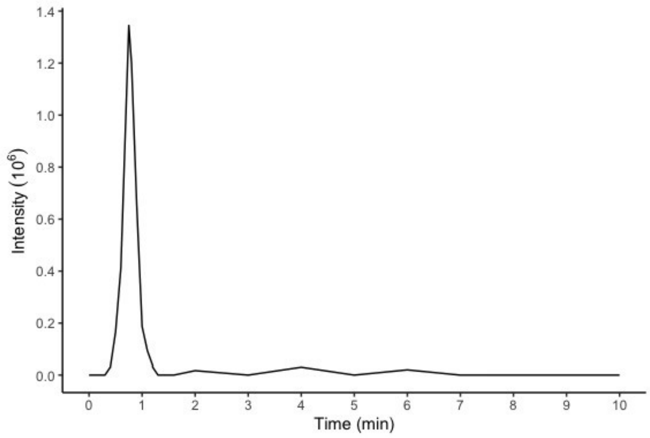

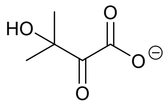

Chemical Formula: $C_5H_7O_4^-$
Exact Mass: 131.0350

Figure S9. UHPLC-HR-ESI-MS analysis of an ion corresponding to the chemically synthesized **3** (*m*/*z* 131.0350 [M-H).

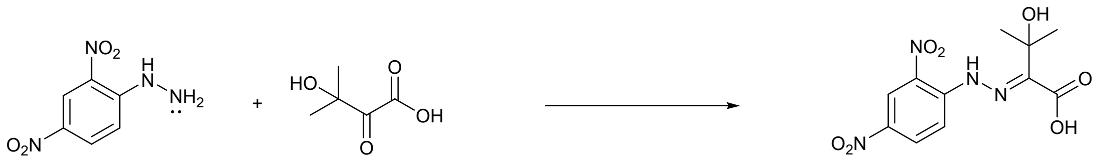

DNPH **3** **HMKB -DNPH**

Figure S10. Derivatization of **3** using 2,4-dinitrophenylhydrazine (DNPH) as the derivative agent. [5]

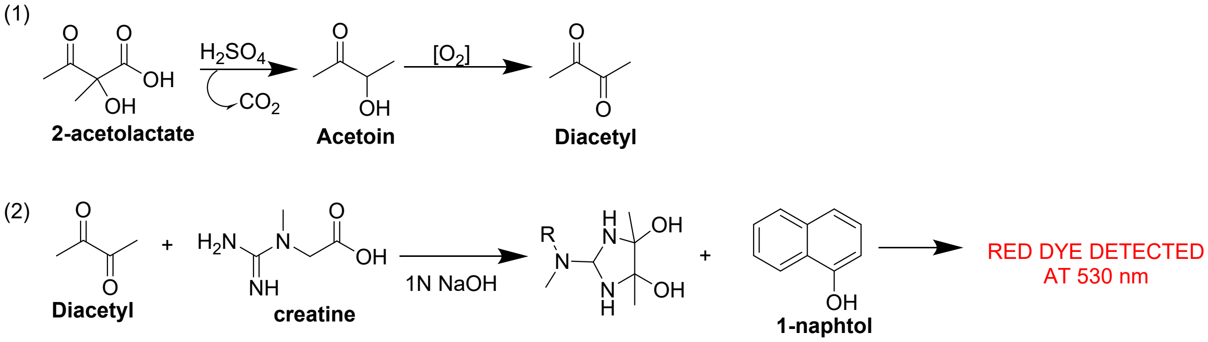

Figure S11. A colorimetric assay of the presence of acetolactate. (1) Decarboxylation of acetolactate under the acidic conditions to form acetoin, followed by spontaneous oxidation of diacetyl. (2) diacetyl reacts with creatine and 1-naphtol to form the red dye which can be monitored at 530 nm by a UV-Vis spectrometer. [2]

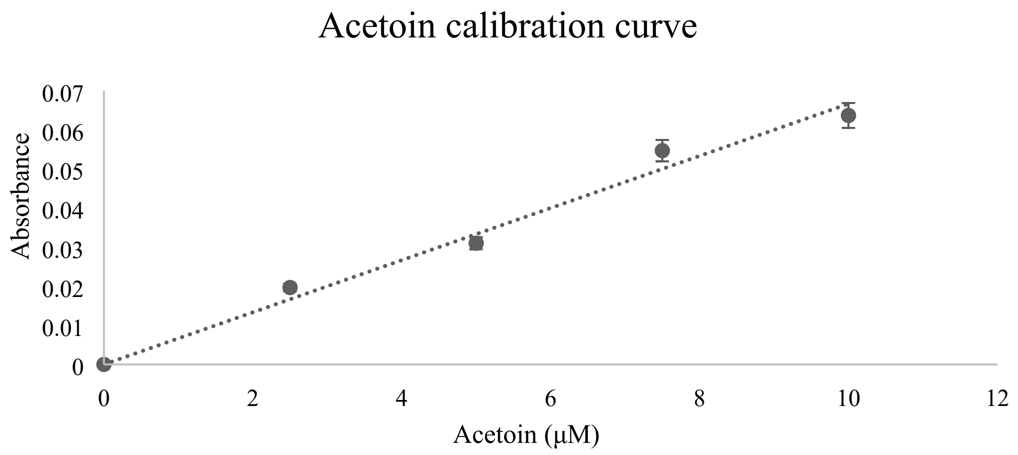

Figure S12. The calibration curve of standard acetoin using the colorimetric assay.

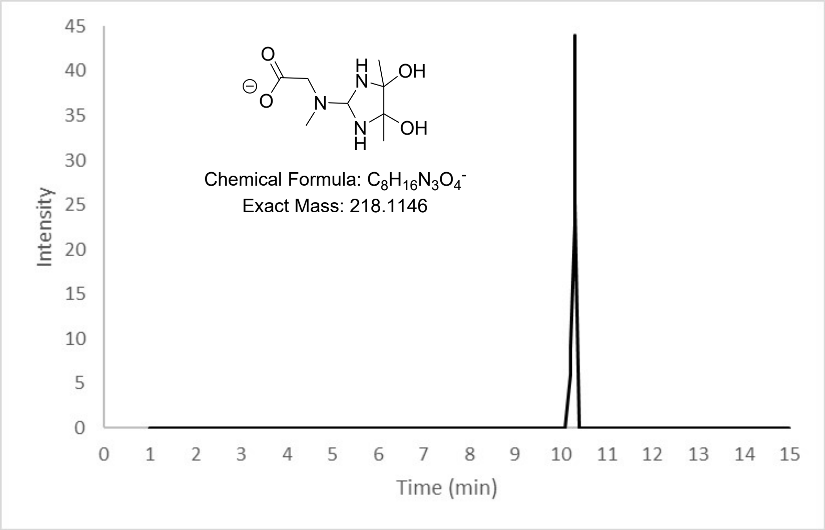

Figure S13. UHPLC-HR-ESI-MS analysis of an ion (*m*/*z* 218.1146 [M$^-$]) corresponding to the heterocyclic product in the acetoin colorimetric assay.

A

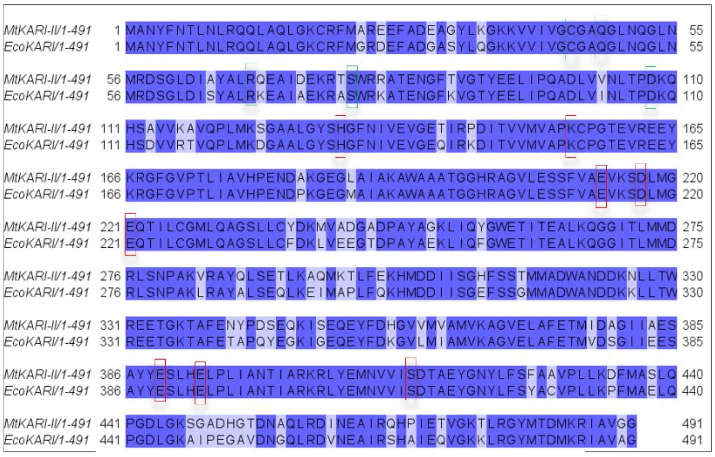

B

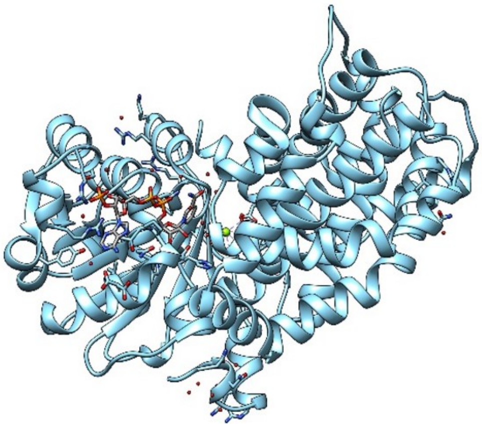

Figure S13. (**A**) Sequence alignment [6] of *Mt*KARI-II and *E.coli* (PDB no. 1YRL) where the green boxes represent the NADPH binding site (C45-Q48-R-68-R76-S78-D108-D113) and the red boxes represent the Acetolactate binding site (H123-K155-E213-D217-E221-E389-E393-S414). (**B**) Protein modelling of *Mt*KARI-II with NADPH and acetolactate bound for homology determination with *E.coli* (PDB no. 1YRL), showing a 100% confidence and a 86% identity.

4. Supplementary table

Table S1. Steady-state parameter comparison comparison between *Mt*KARI-II and other characterizated KARIs from different microorganisms.

Microorganism	NADPH kinetics	NADH kinetics
*MtKARI-II**	k_{cat} = 74±0.2 s^{-1}<br>K_M = 59.03 ± 0.14 µM	k_{cat} = 46.6 ± 3.8 s^{-1}<br>K_M = 318.72 ± 42 µM
*Escherichia coli (strain K12)** [7]	k_{cat} = 7.2 min^{-1}<br>K_M = 0.04 mM	k_{cat} = 0.11 min^{-1}<br>K_M = 0.206 mM
Mycobacterium tuberculosis [8]	K_M = 7.7 µM<br>k_{cat} = 1.4 s^{-1}	Not measured.
Slackia exigua (strain ATCC 700122 / DSM 15923 / CIP 105133 / JCM 11022 / KCTC 5966 / S-7) [9]	k_{cat} = 0.8 s^{-1}<br>K_M = 1 µM	k_{cat} = 0.41 s^{-1}<br>K_M = 45 µM
Lactococcus lactis subsp. lactis (strain IL1403) [9]	k_{cat} = 0.8 s^{-1}<br>K_M = 13 µM	k_{cat} = 0.1 s^{-1}<br>K_M = 285 µM
Metallosphaera sedula (strain ATCC 51363 / DSM 5348 / JCM 9185 / NBRC 15509 / TH2) [10]	k_{cat} = 0.07 s^{-1}<br>K_M = 31 µM	k_{cat} = 0.06 s^{-1}<br>K_M = 24 µM
Archaeoglobus fulgidus (strain ATCC 49558 / VC-16 / DSM 4304 / JCM 9628 / NBRC 100126) [10]	k_{cat} = 0.04 s^{-1}<br>K_M = 26 µM	k_{cat} = 0.1 s^{-1}<br>K_M = 5 µM
Hydrogenobaculum sp. (strain Y04AAS1) [10]	k_{cat} = 0.12 s^{-1}<br>K_M = 46 µM	k_{cat} = 0.12 s^{-1}<br>K_M = 39 µM
Syntrophomonas wolfei subsp. wolfei (strain DSM 2245B / Goettingen) [10]	k_{cat} = 0.22 s^{-1}<br>K_M = 44 µM	k_{cat} = 0.28 s^{-1}<br>K_M = 57 µM
Thermacetogenium phaeum (strain ATCC BAA-254 / DSM 26808 / PB) [10]	k_{cat} = 0.25 s^{-1}<br>K_M = 40 µM	k_{cat} = 0.46 s^{-1}<br>K_M = 1 µM
Corynebacterium glutamicum [11]	k_{cat} = 13.05 min^{-1}<br>K_M= 164 µM	k_{cat} = 0.16 min^{-1}<br>K_M= 53 µM

KARI with stars (*) belong to the Class II KARIs family, the rest are from Class I KARIs.

5. References

1 Tyagi R, Lee Y, Guddat LW, Duggleby RG, *The FEBS Journal*,2005,**272**,593-602.

2 Huo Y, Zhan Y, Wang Q, et al. *Bioprocess Biosyst Eng*. 2018,**1**,87-96.

3 Verdel-Aranda K, López-Cortina ST, Hodgson DA, Barona-Gómez F, *Microb Biotechnol*,2015,**8**,239-252.

4 Squibb and Sons LLC. US Patent.US4973747A, 1990.

5 Uchiyama S, Inaba Y, Kunugita N, *Journal of Chromatography B*, 2011, **879**,1282-1289.

6 Kelley LA, Mezulis S, Yates CM, Wass MN, Sternberg MJE, *Nature Protocols*,2015,**10**,845-858.

7 Chunduru SK, Mrachko GT, Calvo KC, *Biochemistry*,1989,**28**,486-493.

8 Lv Y, Kandale A, Wun SJ, McGeary RP, Williams SJ, Kobe B, Sieber V, Schembri MA, Schenck G, Guddat LK, *FEBSJ*,2016,**283**,1184-1196.

9 Brinkmann-Chen S, Flock T, Cahn JKB, Snow CD, Brustad EM, McIntosh JA, Meinhold P, Zhang L, Arnold FH, *PNAS*, 2013, **110**,10946-10951.

10 Brinkmann-Chen S, Cahn JKB, Arnold FH, *Metab Eng*,2014,**26**,17-22.

11 Lee D, Hong J, and Kim JK. *J. Agric. Food Chem*, 2019, **67**, 8527−8535
